# Appendicitis

**Published:** 1903-03-07

**Authors:** 


### APPENDICITIS.

The results of considerable experience and personal observation of appendicitis are put forward by Dr. MacDougall.[1] The subject is treated mainly from a clinical standpoint and many illustrative cases are described. He states at the outset that he is one of those who believe that appendicitis is largely on the increase both in frequency and severity, and he also considers that relapses are much more common now than formerly. In support of these views he brings forward opinions based on his own experience, and gives a few figures from hospital reports. On the whole we are left much in the same position as we were before with regard to these matters. He points out that it is a disease which runs in families, and quotes several instances of this which have occurred under his own observation; in one family where father and mother had both suffered from appendicitis, two daughters out of four had the disease, suppuration occurring in one case and gangrene in the other. It may be accepted as a fairly safe rule that the acuter the initial symptom is, the greater will be the gravity of the case, and this will be emphasised, Dr. MacDougall thinks, if a rigor occurs accompanying or immediately preceding the acute abdominal pain. Each time he has met with this its occurrence has been associated with a gangrenous appendix. Pain, although it may be referred at first to a place remote from the local trouble, after a variable time is usually localised to the right iliac region. With regard to the value of the pulse as a guide, a frequent *small* pulse—for its character is of greater value than its rapidity—with a high or, worse still, with a low temperature should attract immediate attention, and in closely watching its behaviour—its tendency to quicken or to become slower, to become feebler or to gain in force—the surest helps are to be found, but it must be borne in mind that a suppurative peritonitis may be in progress with a fairly good pulse and a normal temperature. Very high temperature in the beginning with a rapid small pulse and notable *local* conditions demand quick interference. A pulse rising in frequency is an indication for operation. Dr. MacDougall lays considerable stress on the facial aspect of the patient as an indication for interference or for waiting. Costal respiration, especially in the male, is of significance. The presence of a tumour in the right iliac fossa is as characteristic of the majority of cases of acute appendicitis as is the sausage-shaped lump in intussusception, but in the very acute forms there is no lump because it has either not had time to form, or, owing to the existing vital condition, would never form at all. Its presence does not necessarily indicate surgical interference, for such lumps may completely disappear spontaneously. In discussing the diagnosis, Dr. MacDougall remarks that rectal examination should never be neglected. It may confirm the diagnosis where the inflamed organ lies in the pelvis; it may demonstrate an acutely tender mass high up in the iliac fossa; it will certainly show an inflamed peritoneum when external abdominal tenderness may be slightly marked, and it may lead to the recognition of certain conditions that will alter entirely the aspect of the case. A very interesting case of "appendicular colic" is mentioned where very marked collapse indeed following a sudden onset of acute abdominal pain was found at the operation which ensued to be due to a grain of barley in its husk lying transversely in the appendix, which was not perforated. With reference to treatment Dr. MacDougall holds that early operation should be reserved for the grave cases only, and he prefers an incision made nearer to the anterior superior iliac spine than is usual. The use of opium previous to operation is to be avoided if possible as it masks important symptoms; if it must be given, opium administered by suppository is better than morphia by subcutaneous injection. If movement of the bowels is called for enemas are indicated; aperients given by the mouth in these cases are fraught with danger. In the treatment of bad cases where the evidences of peritonitis do not subside after operation, subcutaneous injections of normal saline solution are of the greatest value, and may save lives which might otherwise be lost.

[1] Lancet, Feb. 21.

